# A randomized, controlled, prospective trial to evaluate the haemostatic effect of Lyostypt versus Surgicel in arterial bypass anastomosis: "COBBANA" trial

**DOI:** 10.1186/1745-6215-10-91

**Published:** 2009-09-29

**Authors:** Petra Baumann, Hardy Schumacher, Johannes Hüsing, Steffen Luntz, Hanns-Peter Knaebel

**Affiliations:** 1 Aesculap AG, Department of Clinical Science, Am Aesculap Platz, 78532 Tuttlingen, Germany; 2 Klinikum Hanau GmbH, Department of Vascular Surgery, Leimenstrasse 20, 63450 Hanau, Germany; 3 Coordinating Centre for Clinical Trials (KKS) Heidelberg, Voßstr.2, 69115 Heidelberg, Germany

## Abstract

**Background:**

The development of suture hole bleeding at peripheral arterial bypass anastomoses using PTFE graft prostheses is a common problem in peripheral vascular surgery. Traditionally the problem is managed by compression with surgical swabs and reversal heparin or by using several haemostatic device (e.g. different forms of collagen, oxidized cellulose, gelatine sponge, ethylcyanoacrylate glue or fibrin) with various success. Preclinical data suggest that the haemostatic effect of collagen is stronger than that of oxidized cellulose, but no direct clinical comparison of their hemostatic performance has been published so far.

**Design:**

This randomized, controlled, prospective trial evaluates the haemostatic effect of Lyostypt versus Surgicel in arterial bypass anastomosis. 28 patients undergoing an elective peripheral vascular reconstruction due to peripheral vascular disease will be included. Suture hole bleeding occurring at the arterial bypass anastomosis using a PTFE prostheses will be stopped by the application of Lyostypt and/or Surgicel. The proximal anastomoses will be randomized intraoperatively. The patients will be allocated into 4 different treatment groups. Group1 Lyostypt distal/Surgicel proximal; Group 2: Lyostypt proximal/Surgicel distal; Group 3: Surgicel distal and proximal; Group 4: Lyostypt distal and proximal. Primary endpoint of the study is time to haemostasis. Secondary endpoints are the number of intraoperatively used haemostatic devices, postoperative mortality within 30 days as well as the intraoperative efficacy rating of the two devices evaluated by the surgeon. As a safety secondary parameter, the local and general complication occurring till 30 ± 10 days postoperatively will also be analysed. After hospital discharge the investigator will examine the enrolled patients again at 30 days after surgery.

**Discussion:**

The COBBANA trial aims to assess, whether the haemostatic effect of Lyostypt is superior to Surgicel in suture hole bleedings of arterial bypass anastomoses.

**Trial registration:**

NCT00837954

## Background

### Rationale

Haemostasis in peripheral vascular surgery is made more difficult by the need for direct arterial and arterial graft suturing as well as by systemic anticoagulation to prevent thrombosis during periods of vascular occlusion. Polytetrafluorethylene (PTFE) is one of the most frequently used graft materials for vascular replacement or bypass in the case when no autologous venae are available [1]. However, the insufficient elasticity of PTFE and its porosity promote the development of suture hole bleeding [2,3] which can cause considerable loss of blood and prolongation of operations due to additional suturing with danger of iatogenic stenosis [2].

There are several medical risk factors that can affect bleeding time and haemostasis, such as hypertension, chronic liver disease, or renal failure [4]. The problem of bleeding from suture holes remains a serious drawback to the use of PTFE. Suture line bleeding in arterial surgery is primarily controlled with precise suture technique, including use of fine suture material and needle [5]. To assist with control of residual bleeding after suturing of arterial operative sites, various topical haemostatic aids are in use. Traditionally the problem of suture hole bleeding is managed by compression with surgical swabs and reversal heparin. Other attempts to control suture hole bleeding have also been used with various success, such as ethylcyanoacyrlate glue [6], different forms of collagen [7-10], oxidized cellulose [9,11], or gelatine sponge [9] or fibrin [12,13]. Another approach to topical haemostasis is the use of an agent that enhances or accelerates formation of autogenous thrombus, such as topical thrombin, which can be used in conjunction with scaffolding-type agents [14,15].

In the COBBANA trial the collagen based haemostat Lyostypt will be compared with Surgicel which is the leading standard and is commonly used for this indication.

### Purpose

This study is designed to demonstrate the superiority of Lyostypt to oxidized cellulose (Surgicel) for haemostasis of suture hole bleeding in arterial bypass anastomoses after vascular reconstruction. Lyostypt is an absorbable, wet stable collagen compress made of collagen fibrils of bovine origin. Collagen leads to thrombocyte adhesion and to activation of coagulation factor XII. Therefore collagen is very effective in haemostasis. Collagen is cell-friendly whereas other haemostats significantly disturb cell growth. Advantages of collagen fleece are fast induction of haemostasis, low tissue reaction and fast absorption [16]. Furthermore, collagen was shown to be the best overall haemostatic agent in microvascular surgery. Authors concluded that collagen fleece establish faster haemostasis than oxidized cellulose and that it was resorbed faster than oxidized cellulose [16].

### Need for the study

The ideal haemostat should be easy to use with good risk-benefit and cost-benefit ratios. Such an agent should be easily applied in a controlled fashion, highly predictable in creating haemostasis, nontoxic and must not have an adverse affect on anastomostic patency, increased anastomotic strength would also be beneficial.

Preclinical data strongly suggest that haemostats made of collagen have a stronger haemostatic effect than haemostats made of oxidized cellulose [16]. While both investigational devices have been in clinical use for many years [7-11], no direct clinical comparison of their haemostatic performance has been published so far.

## Methods/Design

### Objectives of the study

The aim of this study is to evaluate the haemostatic effect of Lyostypt and Surgicel in arterial bypass anastomosis. The measurement of the bleeding time of suture hole bleeding of arterial bypass anastomoses is considered as a suitable efficacy parameter for the assessment of the haemostatic effect. It is expected that the treatment of suture hole bleeding of arterial bypass anastomoses with Lyostypt will reduce the time to haemostasis in comparison to the treatment with Surgicel (primary objective). Local complication and general complication rates occurring till 30 ± 10 days after surgery and handling characteristics of the different devices will also be assessed (secondary objectives).

### Primary endpoint

The primary endpoint of the trial is the time to haemostasis (period from release of the cross-clamping to the moment the wound is completely dry or at least sufficiently dry to finalise the operation without additional measures for haemostasis as judged by the surgeon). If haemostasis is not achieved within 3 minutes, a second equal device is applied for another 2 minutes. If haemostasis is still insufficient, the surgeon will wait for another 5 minutes, before considering other methods. When haemostasis is not achieved after 10 minutes, it is considered a study failure.

### Secondary endpoint

- Local and general complication rate till 30 ± 10 days after surgery

- The number of haemostatic devices used

- Intraoperative efficacy rating of each device, (surgical handling characteristics, tissue adhesion of each device)

- Postoperative mortality until 30 days after surgery

- Recurrence of bleeding at the anastomosis,

- Analysis of adverse events

### Design

This is a randomized, controlled, prospective study to evaluate the haemostatic effect of Lyostypt versus Surgicel in arterial bypass anastomoses.

**Figure 1:**
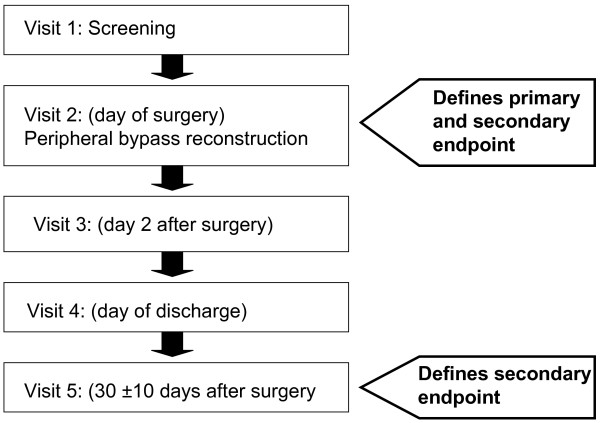
**COBBANA Trial: Schedule of the visits**. This figure represents a flow chart of all visits conducted in the COBBANA trial. The figure also indicates when the primary and secondary endpoints are collected.

In this study, 28 eligible patients with a peripheral arterial bypass following peripheral vascular reconstruction, will be treated with the investigational devices during the pilot phase. There will be 4 treatment groups: Group 1 and 2 will receive both products one at the distal and the other at the proximal anastomosis. In group 3 and 4 the same haemostat will be applicated at both anastomoses either Lyostypt or Surgicel. The proximal bypass anastomosis will be randomized for the application of the haemostat. After discharge from the hospital the investigator will examine the patients at 30 ± 10 days after surgery (figure 1 and table 1).

**Table 1:** Flow Chart COBBANA Trial.

	**Visit 1 (Screening)**	**Visit 2 (OP)**	**Visit 3 (2 ± 1 days post OP)**	**Visit 4 (Day of discharge)**	**Visit 5 (30 ± 10 Days post OP)**
Patient information	X				

Informed consent	X				

Demographic data*	X				

Smoker/non-smoker	X				

Inclusion/exclusion	X				

Past medical history	X				

Physical examination	X	X	X 1)	X 1)	X 1)

Surgery		X			

Time to obtain hemostasis		X			

Deviation from surgical procedures as described in the protocol		X			

Number of used devices		X			

Intraoperative handling of the devices		X			

Recurrence of bleeding		X	X	X	x

Adverse Event/Serious Adverse Event		X	X	X	X

Local and general complication/re-operation		X	X	X	X

Mortality					X

* date of birth, gender, weight, height,

1) of the operational wound

By employing a sequential adaptive statistical design, the results of the pilot phase will be analysed and the appropriate total number of cases determined in a second phase (see section on sample size). If the second phase of this study will be conducted, patients will be recruited and treated as already described for the pilot phase.

### Eligibility

Detailed inclusion and exclusion criteria are specified in table 2. Patients are screened consecutively for eligibility in the participating centre after approval of the study protocol by the local ethics committee. A contract has been signed by Aesculap AG and the participating centre for correct conduction of the trial according to Good Clinical Practice. The participating surgeons performing the intervention have been instructed by detailed manuals.

**Table 2:** Eligibility Criteria.

**INCLUSION CRITERIA**	**EXCLUSION CRITERIA**
• Patients > 18 years	• Emergency surgery
• Informed Consent	• Patients with coagulopathy or uremia
• Indication for a peripheral vascular reconstruction due to peripheral vascular disease	• Participating in another trial with interfering endpoints
• Suture hole bleeding of peripheral arterial bypass anastomosis using PTFE graft	• Patients requiring continuous postoperative anticoagulation
	• Reoperation within one month at the same location
	• Pregnant and breastfeeding women
	• Known or suspected allergies or hypersensitivity to any of the used devices
	• Severe comorbidity (ASA >4)
	• Life expectancy less than 12 months
	• Current immunsuppressive therapy
	• Chemotherapy within last 4 weeks
	• Severe psychiatric or neurologic diseases
	• Lack of compliance
	• Drug- and/alcohol-abuse
	• Inability to understand and to follow the instructions given by the investigator

### Consent

The participating centre recruits trial patients from its patients who are scheduled for an elective implantation of PTFE graft for peripheral revascularization, who develop suture hole bleeding of arterial bypass anastomoses following peripheral vascular reconstruction. All patients who seem to fit to the in- and exclusion criteria will be asked whether they are willing to participate in the COBBANA trial and they will be informed about the purpose of the trial, the operation modalities, data management, and their possibilities and risks. Interested patients will be screened according to inclusion and exclusion criteria and included into the trial after written Informed Consent has been obtained from the patient.

### Randomization

**Figure 2:**
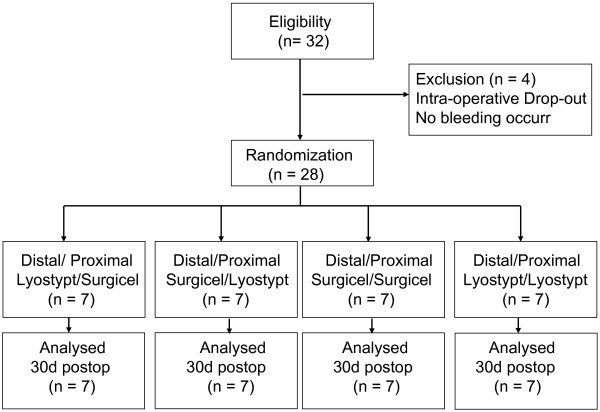
**COBBANA Trial: CONSORT Flow Chart**. Flow Chart of patients according to CONSORT.

Upon inclusion in the study the patient will receive a unique identification number. Intraoperatively their anastomoses will be randomized to receive either Lyostypt or Surgicel by opening sealed opaque envelopes containing the information which device should be used. The combinations proximal and distal: Lyostypt, proximal: Lyostypt/distal: Surgicel, proximal: Surgicel/distal: Lyostypt, proximal and distal: Surgicel will occur in equal quantities (figure 2).

The envelopes will be prepared by the sponsor with balanced distribution of either Lyostypt or Surgicel according to a randomization list provided by the statistician. The randomization list is sealed and kept under lock and key until data queries are resolved and the data base is locked. The envelopes will be assigned to the patients in a chronological order. The envelope contains the devices and also two labels with a randomisation number and the information about the device which was applied to the proximal and distal anastomosis, which must be attached to the CRF and the surgery form.

### Intervention

In the current trial the surgical procedure is standardised to achieve a better comparability of the outcome. For each surgical intervention a Polytetrafluoro-ethylene vascular prostheses from the same manufacture (PTFE prostheses, thin walled, diameters 4-10 mm) will be surgical implanted in accordance with the standard practice of the investigative centre using a non-absorbable polypropylene suture material USP 5-0, USP 6-0 for the anastomotic suture line. The investigator will perform the distal anastomosis first. The clamp will be opened to check for suture hole bleeding. If bleeding occurs, the clamp will be closed and a randomization envelope containing the study material will be opened. The devices will be cut into half. Thereafter, the clamp at the distal anastomosis will be removed and a stopwatch will be started and the randomized device will be placed circumferentially around the anastomosis and applied for three minutes. The suture line is monitored by the investigator and the time required to achieve complete sealing is recorded with a stopwatch. If haemostasis is not achieved after 3 minutes a new equal device is placed around the anastomosis for another 2 minutes and the time to obtain haemostasis is measured with a stopwatch. In the case that suture hole bleeding has not stopped after 5 minutes the investigator will wait for another 5 minutes to achieve haemostasis. Subjects are considered treatment failures if suture hole bleeding could not be controlled within 10 minutes or if additional interventions are required to achieve sealing. Every application of a new device will be recorded with type of device and time after clamp removal.

If haemostasis is achieved at the distal anastomosis the surgeon will perform the proximal anastomosis. Following the anastomotic procedure the clamp at the prosthesis is released. If suture hole bleeding is present, the clamp will be closed. The device according to the randomization procedure will be prepared (if no bleeding at the distal anastomosis had occurred, the randomization envelop has to be opened) and the clamp will be removed and a stopwatch will be started. The randomized device will be placed circumferentially around the proximal anastomosis and applied for three minutes. If haemostasis is not achieved after 3 minutes a new equal device is placed around the anastomosis for another 2 minutes and the time to obtain haemostasis is measured with a stopwatch. In the case that suture hole bleeding has not stopped after 5 minutes the investigator will wait for another 5 minutes to achieve haemostasis. Subjects are considered treatment failures if suture hole bleeding could not be controlled within 10 minutes or if additional interventions are required to achieve sealing. Every application of a new device will be recorded with type of device and time after clamp removal. After achieving haemostasis at the proximal anastomosis the proximal clamp will be removed and the surgical procedure will be finished.

### Study Device

The investigational products are the haemostats Lyostypt and Surgicel (= Tabotamp). For Lyostypt, the size 5 cm × 8 cm and for Surgicel, the size 5 cm × 7,5 cm will be supplied to the study centre.

### Lyostypt

Lyostypt is an absorbable wet-stable collagen compress made of collagen fibrils of bovine origin. Collagen leads to thrombocyte adhesion and to activation of coagulation factor XII. Lyostypt collagen haemostatic fleece is indicated for capillary bleedings, parenchymal haemorrhages, oozing wound haemorrhages, for the local haemostasis in haemodialysis and as a supportive method for other techniques in haemostasis. Lyostypt achieves haemostasis very swiftly, it can be removed easily, it can be applied endoscopically and it can be combined with fibrin glue and antibiotics (16). Collagen haemostats showed low tissue reaction, fast resorption and good biocompatibility (15). Lyostypt is y-sterilized.

### Surgicel/Tabotamp

Surgicel absorbable haemostat is a sterile absorbable knitted fabric prepared by the controlled oxidation of regenerated cellulose. The fabric is white with a pale yellow cast and has a faint caramel-like aroma. It is strong and can be sutured or cut without fraying. After Surgicel has been saturated with blood, it swells into a brownish or black gelatinous mass which aids in the formation of a clot, thereby serving as a haemostatic adjunct in the control of local haemorrhage. When used properly in minimal amounts Surgicel haemostat is absorbed from sites of implantation with practically no tissue reaction.

Surgicel is used adjunctively in surgical procedures to assist in the control of capillary, venous and small arterial haemorrhage when ligation, suturing or other conventional methods of control are impractical or ineffective.

### Sample size

The study is planned in a two-step design. Initially, 28 patients will be enrolled and a test at the α_1 _= .0102 will be performed. The exact number will depend on the number of bleeding anastomoses, as patients will be accrued until 56 applications of a randomized device have been recorded. The main test will be carried out as a parameter test in the model outlined in section statistical analysis.

The trial will be stopped for rejection of the null hypothesis if the one-sided p-value is lower than α_1_, and stopped for futility if it is higher than .50. The level of significance for the final analysis is set to α_2 _= 0.00380/p_1_, where p_1 _is the p-value corresponding to the one-sided test of the primary criterion from the interim analysis, following the approach of Bauer and Köhne [17]. For the second part of the trial, the sample size will be recalculated and will lie between 12 and 72.

This procedure maintains the overall significance level of 2.5 per cent and a power of the one-sided test as described in statistical analysis at 80 per cent. This result was obtained by simulation based on 300 replicates.

### Statistical Analysis

The primary criterion is the time to haemostasis at an anastomosis. For the evaluation of the primary criterion, the single anastomosis is treated as the observational unit. Time to haemostasis, which will be censored at 10 minutes, will be included as the response parameter in an accelerated failure time regression model [18] assuming a Weibull distribution for bleeding time with the following explanatory parameters included: treatment on anastomosis (Lyostypt vs. Surgicel), treatment on opposite anastomosis (Lyostypt vs. Surgicel), site of anastomosis (proximal vs. distal), and center. Subject will be included as a shared frailty term with lognormal distribution. The formal null hypothesis to be tested at a level of α = 0.025 (spent over two tests carried out in different stages) is: "Anastomosis bleeding time under Lyostypt is at least as long as under Surgicel".

Haemostasis during 5 and 10 minutes, local and general complication rate till 30 ± 10 days after surgery, the number of used haemostatic devices minus one will be modelled in a logistic mixed regression model using the same covariates as for the main model. Subject will enter the analysis as a random effect. If model instabilities occur due to overspecification (signified by a condition number of the design matrix of more than 40), the term "treatment on opposite anastomosis" and possibly the center effect will be dropped.

The intraoperative efficacy rating scale of each device will be modeled separately for the 4 step ranking scale of adhesion and the 5 step ranking scale of handling, using a logistic mixed model of ordinal responses using the same covariates as in the main model as fixed and surgeon and subject as random effects. Postoperative mortality will be described individually in the final report. Adverse events which are recorded will be reported casuistically, mentioning the haemostypt actually applied at the distal and proximal site.

### Interim Analysis

One interim analysis is planned. The number of patients to be included after interim analysis will be calculated based on the p-value of the parameter test in the model for the main parameter outlined above based on the adaptive design proposed by Bauer and Köhne [17]. The interim analysis will be used to test the distributional assumption of a Weibull distribution for the bleeding time (using the Anderson-Darling test statistics for the residuals for a test at the 10 per cent level), in order to perform the analysis on the rest of the patient using a more appropriate distribution.

### Clinical site and safety aspects

Sites are selected according to their experience in peripheral arterial bypass reconstruction, their clinical research experience, to the number of eligible patients operated in the indicated intervention per year and to their willingness to adhere to clinical trial protocol. The participating centre is mentioned at the end of this paper.

As Lyostypt and Surgicel are used locally a systemic effect can be excluded, only adverse events that affect the peripheral arterial bypass region during the end of operation and the last visit of the patient will be documented on the AE form in the CRF. All suspected unexpected serious adverse events and reactions (SUSAR) will be reported from the principal investigator to the sponsor. The sponsor will inform the leading ethical committee about the unexpected SAEs and SUSARs. For safety analysis the incidence of AE and SAE occurring in the peripheral arterial bypass region will be analysed. Patients may withdraw from the study at any time either at their own request or at the request of the principal investigator.

### Trial organization, quality control, registration and ethical aspects

The COBBANA trial is initiated and sponsored by B|Braun Aesculap and conducted by Aesculap AG. The Coordinating Centre of Clinical Trials (KKS) in Heidelberg is responsible for the data management and the biometrics. Monitoring is done by a independent person of Aesculap AG who will visit the investigational site at regular intervals to verify adherence to the protocol and perform source data verification. Aesculap AG is responsible for the management and the registration (Identifier Number NCT00837954, ). Enrolment must be reported immediately by the investigator by fax on prepared forms to the sponsor. Quality assurance will be done in cooperation with the KKS in Heidelberg. The trial is performed according to the Declaration of Helsinki in its current German version and the Good Clinical Practice (GCP). Before the start of the trial the independent ethics committee of the Landesärztekammer Hessen gave their positive vote on 3^rd ^February 2009. The study was registered on 5^th ^February 2009.

### Current status and duration of the trial

The study protocol for the trial was completed on 5^th ^November 2008. Following completion of the contract the study was initiated on 28^th ^January 2009. Currently one centre recruits patients with the goal of 28 patients. After receiving the positive ethics vote the first patients was randomized on 26^th ^February 2009. Assuming an overall enrolment of 3 patients per month, the end of recruitment is expected for October 2009.

## Discussion

For topical haemostasis bone wax, and various other topical haemostatic agents (powdered oxidized cellulose in polyethylene glycol wax, oxidized cellulose, gelatine paste, microfibrillar collagen, gelatine sponge soaked in thrombin) are currently in use [19]. Haemostasis is achieve through the physical, mechanical (bone wax, gelatine, oxidized cellulose) and chemical effect (microfibrillar collagen) of these materials [15,19]. Bone wax is non-absorbable whereas the other haemostatic agents are absorbed within days and weeks. Complications like allergic reactions, infections, granulomas and interference with bone healing have been noticed only rarely after the application of theses materials [15].

Only a few controlled clinical trials exist that compare two or more haemostatic agents [9,20-27]. Voormolen et al. [28] conducted an experimental study in rabbits in which cerebral lesions were made and filled with oxidized regenerated cellulose and collagen fleece. Results showed lower bleeding times for microfibrillar collagen with quicker resorption rate than for traditional oxidized regenerated cellulose. They concluded that collagen fleece established faster haemostasis and that it was faster resorbed than oxidized cellulose. Rybock et al. [29] compared the haemostatic properties of microfibrillar collagen versus a gelatinfoam in suction-evacuation lesions of the canine cortex. Microfibrillar collagen was found to be faster and more effective than the gelatine foam in achieving haemostasis. Wagner et al. [30] quantitatively compared six commonly used topical haemostatic agents in terms of their ability to mediate platelet aggregation, deposition and activation in a series of in vitro tests. They presented an overall activity ranking of the materials used: collagen >gelatine>oxidized regenerated cellulose. Finn et al. [31] concluded that microfibrillar collagen, oxidized regenerated cellulose, and gelatine foam might be adequately used in iliac procurement, whereas bone wax seems to be contraindicated. Microfibrillar collagen was showed to be the best overall haemostatic agent in microvascular surgery [32]. The prospective randomized neurosurgical trial reported by Krüger et al. [25] showed superior haemostatic effect of a collagen fleece (Lyostypt) compared with gelatine foam.

Suture hole bleeding in patients undergoing vascular reconstruction with polytetrafluoroethylene grafts is a considerable problem in vascular surgery. Various animal and clinical studies evaluating the haemostatic efficacy of different materials demonstrated that fibrin sealant is very efficient [12-14,33,34],. Comparison of fibrinogen or thrombin coated collagen material with surgical swabs indicated a reduction of blood lost and time to haemostasis when the collagen agent was used [10,11,35,36]. These data suggest that fibrin sealant is very efficient in the haemostasis of suture hole bleeding but it is expensive and made from human blood which maybe interfere with incompatibility and may have the risk of HIV virus and Hepatitis A contamination. Therefore an effective, absorbable, easy to use, medically safe and economic material to provide fast local haemostasis after arterial bypass anastomosis is demanded. Oxidized regenerated cellulose and fibrillar collagen are very useful.

Oxidized cellulose seems to confer haemostasis by decreasing the pH and acting as a caustic, thus generating an artificial clot. The clot is brownish because of the production of acid haematin. Advantage of oxidized cellulose is its definite and potent action against a wide variety of pathogenic organisms due to its low pH. Oxidized cellulose does not promote platelet aggregation.

The efficacy of collagen-derived haemostatic agent has been established in standardized animal studies [9,37-41] and clinically in man [20,25,42-44]. Collagen induces haemostasis in two ways, first it activates the aggregation of thrombocytes and secondly it invokes factor XII (Hagemann Factor) which promotes the formation of a fibrin clot. Advantages of collagen fleece are the fast induction of haemostasis, low tissue reaction fast absorption and the possibility that excess collagen can be carefully teased away from around the haemorrhage site without re-initiating bleeding.

Several authors [20,25,37] have shown the effectiveness and clinically safety of the collagen fleece, Lyostypt for topical haemostasis but a direct comparison with oxidized regenerated cellulose in respect to stop arterial suture hole bleedings have never been done before.

Therefore COBBANA the first randomized controlled trial, comparing the haemostatic effect of a collagen based haemostat (Lyostypt) versus an oxidized cellulose based haemostat (Surgicel) in arterial bypass anastomosis, was designed to generate high quality clinical evidence. If significant, the results might be generalized and may change the current treatment practice of suture hole bleedings.

The choice of a one-sided test in lieu of a two sided test is in need of justification. As Surgicel is the standard device used in the treatment of suture hole bleeding, the successful rejection of an additional one-sided hypothesis in the opposite direction (effectively carrying out a two-sided test at the .05 significance level) would lead to the same action as the failure to reject the hypothesis in favour of Lyostypt: Surgicel would remain the product of choice as a haemostypt for anastomoses. As further action would not depend on the outcome of the opposite one-sided test, a one-sided test would be appropriate here. Bland and Bland [45] discuss that " [...] a one sided test is appropriate when a large difference in one direction would lead to the same action as no difference at all." Bauer and Köhne [17] suggest one-sided tests for two reasons: Combining two-sided p-values from two different stages of the trial would defy interpretation if the effects would point in opposite direction, and the option of stopping for futility after the first stage would only be available in the case of results very close to the situation of no effect. Additionally, it is considered a waste of resources and ethically doubtful to carry on the trial if the intermediate results favour Surgicel, and the rest of the trial could only be used to establish the superiority of the standard treatment.

## Conclusion

The COBBANA trial is a randomized controlled prospective study to evaluate the haemostatic effect of Lyostypt versus Surgicel in arterial bypass anastomosis. As clinical relevant conclusion the COBBANA trial aims to find out whether the use of Lyostypt will stop suture hole bleedings significantly faster than Surgicel.

## Competing interests

Aesculap AG, Germany sponsors this study and its publication.

## Authors' contributions

PB and HPK (B|Braun Aesculap, Tuttlingen, Germany) managed and conducted the trial together with HS. PB and HPK wrote together with HS the manuscript. JH and SL are involved in the data management and biometrics. All authors have read and approved this manuscript.
